# Activated Microglia Mediate the Motor Neuron‐, Synaptic Denervation‐ and Muscle Wasting‐Changes in Burn Injured Mice

**DOI:** 10.1002/jcsm.13755

**Published:** 2025-03-04

**Authors:** Jingyuan Chen, Yoshinori Kitagawa, Fei Xie, Haobo Li, William R. Kem, Zerong You, Shingo Yasuhara, J. A. Jeevendra Martyn

**Affiliations:** ^1^ Department of Anesthesiology, Critical Care and Pain Medicine, Massachusetts General Hospital, Shriners Hospital for Children and Harvard Medical School Boston Massachusetts USA; ^2^ Department of Anesthesiology The First Affiliated Hospital, Sun Yat‐Sen University Guangzhou China; ^3^ Division of Anesthesiology and Critical Care Medicine, Department of Surgery Tottori Japan; ^4^ Sichuan Cancer Hospital & Institute, Sichuan Cancer Center, School of Medicine University of Electronic Science and Technology of China Chengdu China; ^5^ Department of Pharmacology University of Florida Gainesville Florida USA

**Keywords:** burn injury, inflammatory cytokines/chemokines, microglia activation, muscle wasting, neuronal apoptosis

## Abstract

**Background:**

Muscle wasting (MW) of burn injury (BI) remains unresolved. Microglia‐mediated inflammatory cytokine/chemokine release, motor neuron loss (MNL) and MW is observed after BI but connection of the central changes to synaptic‐denervation and MW is unelucidated. Stimulation of microglia α7acetylcholine receptors (α7AChRs), a Chrna7gene‐product, exhibits anti‐inflammatory properties (decreased cytokine/chemokines). Hypothesis tested was that exploitation of the microglia α7AChR anti‐inflammatory properties mitigates cytokine inflammatory responses, MNL, synaptic‐denervation and MW of BI.

**Methods:**

Wild‐type or α7AChR knockout (A7KO) mice received 30% body BI or Sham BI (SB) under anaesthesia with and without selective α7AChR agonist, GTS‐21. Lumbar spinal cord tissue and hindlimb muscles were harvested. Immunohistochemistry, TUNEL assay for apoptosis and/or Nissl staining were used to examine microglia (Iba1 staining), MNL (NeuN staining) and synapse morphology (synaptophysin for nerve and α‐bungarotoxin for muscle α7AChR). Spinal cytokine/chemokine transcripts, inflammatory transducer‐protein expression and tibialis, soleus and gastrocnemius muscle weights were measured.

**Results:**

BI to Wild‐type mice caused significant microgliosis (5.8‐fold increase, *p* < 0.001) and upregulated TNF‐α, IL‐1β, CXCL2, MCP‐1 transcripts, and inflammatory transducer‐protein (STAT3 and NF‐κB, *p* < 0.01) expression together with increased transcripts of iNOS (*p* < 0.01) and CD86 (*p* < 0.01) at day 14 reflective of inflammatory M1 microglia phenotype. Significant apoptosis, MNL (32.2% reduction, *p* < 0.05), increased spinal caspase‐3 expression (> 1100‐fold, *p* < 0.05) and synaptic denervation were observed with BI. The tibialis muscle endplates (synapse) of SB had a smooth pretzel shaped appearance with good apposition of presynaptic nerve to postsynaptic muscle. In BI mice, the normal pretzel‐like appearance was lost, and the endplates were fragmented with less nerve to muscle apposition. Tibialis, soleus, and gastrocnemius mass were decreased 31.7% (*p* < 0.01), 23.4% (*p* < 0.01) and 27.5% (*p* < 0.01) relative to SB. The A7KO mice with SB showed significant MNL loss (61.5% reduction, *p* < 0.05), which was aggravated with BI, accompanied by significantly higher expression of STAT3 and Nf‐kB (*p* < 0.05). GTS‐21 ameliorated the spinal expression of above enumerated cytokines/chemokines, inflammatory transducer‐proteins (*p* < 0.05) together with mitigated MNL (*p* < 0.05), synaptic denervation (*p* < 0.05) and decreased MW of tibialis (25%), gastrocnemius (15%) and soleus (20%) relative to untreated wild type BI mice (*p* < 0.01). GTS‐21 beneficial effects were absent in the A7KO mice.

**Conclusions:**

Microglia‐mediated inflammatory responses play pivotal role in MNL as decrease of inflammatory responses improved MNL; α7AChR stimulation also mitigated synaptic denervation and MW changes of BI. α7AChRs have a role in spinal homeostasis even in uninjured state.

**Abbreviations**
α7AChRS: acetylcholine receptors: α7acetylcholine receptors
A7KO: α7acetylcholine receptor knockout
BCA: bicinchoninic acid
BI: burn injury
CXCL2: C‐X‐C motif chemokine ligand 2
DAPI: Diamidino‐2‐Phenylindole
GAPDH: glyceraldehyde‐3‐phosphate dehydrogenase
IHC: immunohistochemistry
IL‐1β: inteukin‐1β,
Iba1: ionised calcium‐binding adapter molecule 1
L3‐L4 segments: lumbar spinal cord
MNL: motor neuron loss
MCP‐1: monocyte chemoattractant protein
NF‐κB: nuclear factor‐kappa B
OCT: optimal cutting temperature
SB‐BI: Sham burn Injury
RT‐PCR: real‐time quantitative polymerase chain reaction
STAT: signal transducer and activator of transcription3
TUNEL: terminal deoxynucleotidyl transferase dUTP nick‐end labelling
TNF‐α: tumour necrosis factor‐α

## 1 Introduction

Burn injury (BI) occurs globally in ~11 million people per year and is the leading cause of injury in children [1]. Despite advances in critical care and survival, the muscle wasting (MW) and neuromuscular dysfunction of BI remains unresolved [2, 3, 4], and have serious public health consequences affecting quality of life, morbidity and mortality even 5 years after BI [5, 6]. Most previous studies, focusing on correcting the aberrant anabolic/catabolic signalling changes and/or use of enhanced nutritional/anabolic supplements, have not successfully rectified the MW of BI [2, 7, 8]. Thus, further understanding of molecular mechanisms of loss of muscle mass or MW of BI is a key area of study that needs novel approaches in order to provide effective corrective therapeutics.

Previous studies from this laboratory have indicated that BI induces a denervation‐like state evidenced as increase in muscle acetylcholine receptors (AChRs), a pathognomonic biochemical feature of denervation [9, 10]. The aetiology of the denervation‐state, whether it is due to factors central or peripheral in origin, has never been elucidated. Both impaired motor neuron to muscle anterograde signalling and peripheral muscle to nerve retrograde signalling can cause synaptic denervation [11, 12]. Even following a small localised (1%) BI, there is a time‐dependent microgliosis (proliferation of microglia) on the dorsal horn of the spinal cord supplying the area of injury [13, 14]. Major body BI also leads to a time‐dependent microgliosis, microglia activation (increased inflammatory cytokine release) in both dorsal and ventral horns together with motor neuron loss (MNL) [15] suggestive of a central nervous system (CNS)‐component to MW but a cause‐and‐effect relationship between the microglia activation and motor neuron changes to MW has not been established.

Microglia are the resident macrophages of the CNS, representing 5–10% of total CNS cells [16]. Microglia, originally thought to be supportive structures, are now confirmed to play a major role in neuro‐pathophysiology [17, 18]. As immune‐competent macrophage‐like cells, the microglia maintain CNS homeostasis by constant surveillance and are activated by pathologic events (e.g., injury or pathogens).

Furthermore, many systemic inflammatory diseases can lead to neuro‐inflammation together with microglia activation [19, 20, 21, 22]. although the typical signs of inflammation, namely edema (*tumour)* and heat (*calor*), are usually absent. Microgliosis and/or activated microglia occurs in conjunction with release of inflammatory cytokines and chemokines, which can modulate function and even survival of neurons as shown in neuropathologic MW states of encephalitis, amyotrophic lateral sclerosis (ALS), and Alzheimer's disease [19, 21, 22]. Thus, normalisation of innate immune microglia dysfunction and associated neuro‐inflammatory responses could benefit MNL and MW of BI.

The α7 nicotinic acetylcholine receptors (α7AChRs), encoded by Chrna7 gene, plays an important role in the brain and innate immune system signalling including macrophages and microglia [23]. The innate immune macrophage and microglia cells constitutively express α7AChRs, which when stimulated exhibit anti‐inflammatory properties and improved pain responses of BI [13, 14]. Hypothesis tested was that microglia‐mediated cytokine and chemokine release plays a pivotal role in the motor neuron changes and distant synaptic disintegration and MW of BI and mitigation of microglia activation by agonist stimulation of α7AChRs attenuates microglia‐mediated inflammatory cytokine/chemokine responses, motor neuron, synaptic (junctional) disintegration/denervation and MW of BI.

## 2 Materials and Methods

### 2.1 Materials and Reagents

RNeasy plus Universal Kit was obtained from Qiagen (Germantown, MD). High‐Capacity RNA‐to‐cDNA Kit, Platinum SYBR Green qPCR Super Mix, ViiA7 Real‐Time PCR System, Pierce 660 nm Protein Assay kit, Secondary antibodies conjugated with Alexa Fluor 488, Alexa Fluor 594, DyLight 680, and DyLight 800 were from Thermo Fisher Life Technologies (Grand Island, NY). ApopTag fluorescein kit and primary antibody against NeuN were from Millipore Sigma (Burlington, MA). Nova Ultra Nissl stain kit was from IHC World (Woodstock, MD). Iba1 antibody was from FUJIFILM Wako Pure Chemical Corporation (Japan), primary antibody against unphosphorylated and phosphorylated signal transducer and activator of transcription3 (STAT, pSTAT3), unphosphorylated and phosphorylated nuclear factor‐kappa B (NF‐κB. pNF‐κB) were obtained from Cell Signalling Technology Inc (Danvers, MA). Alexa Flour 594‐labelled α‐bungarotoxin for labelling of synaptic acetylcholine receptor was from Thermo Fisher Life Technologies (Grand Island, NY). Protease inhibitor Cocktail were obtained from Sigma (St. Louis, MO). The selective α7AChR agonist was synthesised at the University of Florida (Gainesville, FL, William E. Kem, Ph.D.). The pharmacological properties of GTS‐21 including its anti‐inflammatory properties and its brain penetration have been previously described [24, 25]. GTS‐21 was dissolved in physiological saline (pH 6.5), kept refrigerated, administered (4 mg/kg) twice daily I.P.

### 2.2 Animals, Burn Injury Model and Experimental Design

The study protocol was approved by the Institutional Animal Care Committee at Massachusetts General Hospital (Protocol # 2013 N000193). Wild‐type (WT, C57BL6/J) and mice deficient for a7AChR (A7KO) on C57BL6/J background (B6.129S7‐Chrna7tm1Bay, Stock No. 003232) were purchased from The Jackson Laboratory (Bar Harbour, Maine). Mice homozygous for the a7AChR null allele are viable and fertile. Neuropathological and histochemical assessment of brains from α7nAChR knockout mice reveal no abnormalities and have high‐affinity [I‐125] alpha‐bungarotoxin binding sites in the brain. Breeding of homozygous knockout mice or wild‐type mice was established to obtain progenies. All animals (20–25 g body weight) were habituated to environment for 1 week before the experimental procedures. Mice (*n* = 80) were randomly divided into sham‐burn (SB) control or BI group, with or without treatment with selective partial α7AChR agonist, GTS‐21. The lumbar 3–4 spinal cord ventral horn and skeletal muscle tissues were harvested on day 7 and 14 after perturbations.

BI was produced under anaesthesia using a mixture of ketamine (100 mg/kg, body weight) and xylazine (5 mg/kg) mixture given intra‐peritoneally. Third degree BI was produced to achieve a 30% total body surface area injury, by immersing the back side and both flanks of body for 5 s and abdominal side for 4 s in 80°C water, as previously described [15]. Fluid resuscitation was performed by injecting 1 mL of normal saline to both groups. Buprenorphine (0.1 mg/kg, subcutaneously) was provided as analgesia every 8 h for the first 3 days and was administered to both BI and Sham BI mice. The age‐ and weight‐matched SB group were treated in the same manner as the BI group, except that they were immersed in lukewarm water. BI mice were permitted to consume food *ad libitum* and pair feeding of sham‐burned (SB) mice were attempted; no obvious differences in food intake were observed, however, between groups. At day 7 or 14 after BI and treatments, the animals were euthanized and the muscle (gastrocnemius, soleus and tibialis anterior) and spinal cord (Lumbar 3–4 ventral horn) segments were harvested.

### 2.3 Immunofluorescent Staining

Mice were euthanized and tissues were perfuse‐fixed with 4% paraformaldehyde in PBS followed by further immersion fixation in 4% paraformaldehyde in PBS at 4°C for overnight, and subsequently transferred to 15% and 30% sucrose solution. Lumbar spinal cord (L3‐L4 segments) and skeletal muscle tissues were collected and embedded in an optimal cutting temperature (OCT) compound for frozen sections and cut into 10 or 30 μm thick slices. The sections were quenched in 0.1% sodium borohydride and incubated in 1% BSA solution for 1 h at room temperature. They were then incubated overnight with ionised calcium‐binding adapter molecule 1 (Iba1, a microglia marker, 1:300; Wako, Japan) and NeuN primary antibody (neuron marker, 1:300; Merck Millipore, Bedford, MA) for staining spinal cord ventral horn sections. Synaptophysin antibody (Abcam, 1:300) was used for nerve terminals and α‐bungarotoxin (α‐BTX, 1:500, Thermofisher) for AChRs in muscles. The sections were washed with PBS before being incubated with Alexa Fluor 568‐ and Alexa Fluor 488‐conjugated secondary antibodies and mounted in Vectashield mounting medium with 4′,6‐Diamidino‐2‐Phenylindole (DAPI) for nuclei counter‐staining. Images were recorded by Nikon Eclipse microscope E800 and ZEISS LSM800 Confocal microscope.

### 2.4 Nissl Staining

To observe the morphology and number of motor neurons, lumbar L3–4 spinal cord segments were cryo‐sectioned at 10 μm, dehydrated through 100% and 95% alcohol, rehydrated in distilled water, and stained according to Nova Ultra Nissl stain kit protocol (IHC World, Woodstock, MD). Subsequently, the slices were immersed in 95% ethyl alcohol for 5 min, dehydrated in 100% alcohol and cleared in xylene for 5 min. Spinal cord slices were mounted and observed under Nikon Eclipse E800 light microscope. The numbers of motor neurons in the spinal ventral horn (neurons with a diameter ≥ 25 μm) were counted in each animal.

### 2.5 Apoptosis Testing

L3‐L4 segments of lumbar spinal cords were collected and embedded in OCT compound cryosectioned into 10 μm thick slices. Apoptotic cell death was detected using the *in‐situ* terminal deoxynucleotidyl transferase dUTP nick‐end labelling (TUNEL) assay according to the manufacturer's instructions (Millipore, ApopTag fluorescein in situ apoptosis detection kit). Sections were then incubated with NeuN (neuronal marker) primary antibody (1:300; Merck Millipore, Bedford, MA) and Alexa Fluor 568‐conjugated secondary antibody for counter‐staining against neurons. Nuclear‐staining agent, 4′,6‐diamidino‐2‐phenylindole (DAPI) was added and mounted on glass slides. Images were recorded by Nikon Eclipse E800 microscope for the whole tissue reconstitution and the TUNEL/NeuN positive cells were counted in each section at 200x magnification.

### 2.6 Real‐Time Quantitative PCR (RT‐PCR)

To determine if BI‐induced microglia activation results in increased inflammatory responses in the spinal cord, real‐time quantitative PCR was performed to analyse mRNA expression of cytokines and chemokines in lumbar spinal cord segments. Total RNA was extracted using RNeasy plus kit (Qiagen, Germantown, MD) from the ventral horn of the spinal cord. The first strand cDNA was synthesised using RNA‐to‐cDNA Kit (Thermo Fisher Life Technologies) and served as the template for real‐time PCR. Real‐time RT‐PCR reactions were carried out using Platinum SYBR Green RT‐PCR Super Mix (Thermo Fisher Life Technologies) on a 7 ViiA7 Real‐Time PCR System (Life Technologies). Each reaction was performed in duplicate independently with endogenous controls (mouse GAPDH).

### 2.7 Western Blot

Spinal cord (L3‐L4 segment) and gastrocnemius muscles were homogenised in tissue lysis buffer (Sigma, St. Louis, MO) containing protease inhibitors. After centrifugation of homogenates at 12 000 g for 15 min at 4°C, the supernatants were extracted, and the protein concentrations determined using the bicinchoninic acid (BCA) protein assay (Pierce, Grand Island, NY). Equal protein amounts were separated by 4–12% SDS‐PAGE and transferred onto polyvinylidene difluoride membranes. The membranes were blocked in blocking buffer for 1 h, incubated with primary antibodies for glyceraldehyde‐3‐phosphate dehydrogenase (GAPDH) as loading control and pStat3 and pNF‐κB overnight at 4°C. After three washes, the membranes were incubated with fluorescent‐conjugated anti‐rabbit or anti‐mouse antibody for visualization by LiCor Odyssey Imaging System and the detected bands were quantified and normalised to the loading control, GAPDH.

### 2.8 Muscle Weight Measurement

After removing connective tissues and tendons, the harvested skeletal muscle samples were dehydrated for 18 h by incubating at 60°C. Muscle protein weight was then normalised to day 0 (pre‐burn) body weight. This was because after BI, both body and muscle continue to lose mass with time and therefore normalising muscle mass relative to the day of harvesting would not reflect true protein loss.

### 2.9 Statistical Analysis

All data are expressed as the mean ± SD and were analysed using Prism (Graph Pad Software, Inc., La Jolla, CA, USA). Comparisons differences between groups were performed using one‐way ANOVA and subjected to Tukey's multiple comparison tests. A *p*‐value < 0.05 was considered statistically significant.

## 3 Results

### 3.1 Burn Injury Causes Microgliosis, Decreased Neuronal Expression and Apoptosis in Ventral Horn

**FIGURE 1:**
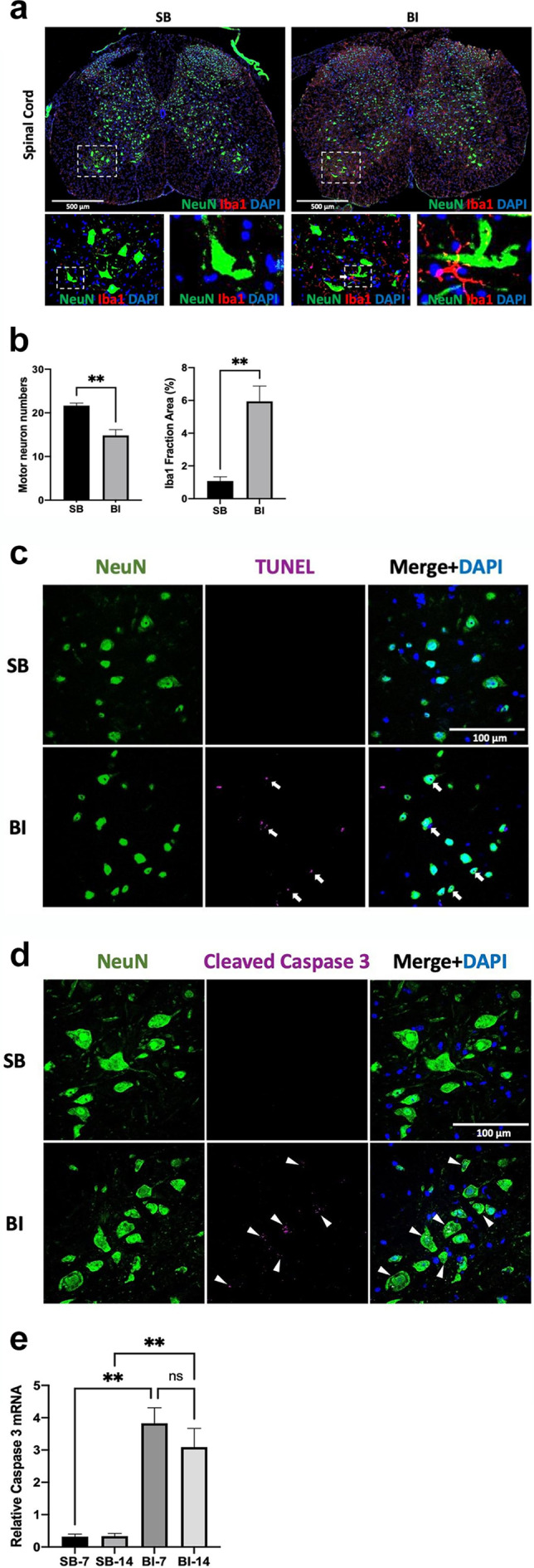
**BI leads to microgliosis together with neuronal apoptosis and decreased neuronal numbers in the ventral horn of spinal cord**. **a**: Confocal images (20X magnification) of triple immunofluorescence staining of L3–4 segments of spinal cord ventral horn with microglia‐marker, Iba1 (red), neuron‐marker, NeuN (green), nuclear‐marker, DAPI (blue) at 14 days after sham‐burn (SB) or burn injury (BI). Motor neurons decreased in numbers and were smaller in size together with prominent proliferation of microglia (microgliosis) at day 14 after BI (inset of dotted white box and magnified at 63x bottom of Figure 1a). Compared to SB, the microglia (red) in BI appeared juxtaposed to the motor neurons (white arrow in magnified image below Figure 1a). **b**: The numbers of neurons were significantly (*p* < 0.001) decreased in the ventral horn area (200x magnification) at day 14 after BI as compared to SB (Figure 1b). (Analysis on the motor neuron numbers used a threshold of 25 μm or higher as a motor neuron). The microgliosis was analysed by intensity of the staining of Iba1 from 5 sections/mouse, 4–6 animals/group. Compared to SB, microgliosis was significant (*p* < 0.001) **c**: In situ TUNEL apoptosis assay (magenta) together with counter‐staining with neuronal maker (NeuN, green) and nuclei (DAPI, blue) of the ventral horn area was performed. As compared to SB, spinal cord of BI showed prominent increased TUNEL positive nuclei in the neurons (magenta). White arrows point to positive magenta staining contained within neurons in the merged Figure 1c. **d**: Immunostaining against activated caspase‐3 (magenta) in the ventral horn area is shown with counterstaining against neurons (NeuN, green) and nuclei (DAPI, blue). The merged figure shows overlap of neurons and capase‐3 (Figure 1d). BI group showed significantly increased level of activated caspase‐3 staining. **e**: RT‐PCR against caspase‐3/GAPDH showed increased level of capase‐3 transcripts in the ventral horn of the spinal cord in the BI mice as compared to SB at day 7 and 14. **p < 0.05 vs. SB group. ns: not statistically significant.

Confocal images (20X magnification) were used after triple immunofluorescence staining of lumbar 3–4 (L3–4) segments of spinal cord ventral horn using microglia‐marker, 1ba1 (red), neuronal maker, NeuN (green), nuclear maker, DAPI (blue) at Day 14 after sham burn (SB) or burn injury (BI). Motor neurons numbers appeared decreased and were smaller in size together with prominent microgliosis at day 14 after BI (inset of dotted white box and magnified at 63X bottom of Figure 1a). (At that magnification, a threshold higher than 25 μm was used to identify motor neuron numbers) [15]. When motor neuron numbers per field were counted, there was a significant decrease in the motor neuron numbers after BI (Figure 1b left). Quantitation of the occupied microglia area revealed a significant dorsal and ventral horn microgliosis in the BI mice (Figure 1b right). Some of the microglia processes (red) in BI injured mice were juxtaposed to the green stained neurons (magnified images in bottom right of Figure 1a). To examine if the MNL was due to apoptosis, in situ TUNEL assay was performed in the ventral horn together with counter staining of neurons (NeuN) and nuclei (DAPI) to quantify the extent of neuronal apoptotic cell death (Figure 1c). Compared to SB, BI mice showed increased TUNEL positive nuclei within the neuron's indicative of neuronal cell death (Figure 1c, white arrows point to positive TUNEL staining within neurons in the merged figure indicative of neuronal apoptosis). The spinal cord sections were also immunofluorescent‐stained against activated cleaved caspase‐3, a pivotal enzyme causing apoptotic cell death (Figure 1d). BI mice showed significantly increased of caspse‐3 at day 14; (Figure 1d). The merged figure shows that the increased caspace‐3 activity was within the neuron. RT‐PCR of caspase‐3 normalised to GAPDH (internal control) showed significantly increased transcripts in the ventral horn of BI mice at day 14 (Figure 1e). Overall, these immunofluorescent staining data indicate significant microgliosis, increased apoptosis, and decreased motor neuron numbers together with caspase‐3 mediated apoptosis of neuronal death in the ventral horn of BI mice.

### 3.2 BI Leads to Microglia Inflammatory Cytokine Release in the Spinal Cord Ventral Horn

**FIGURE 2:**
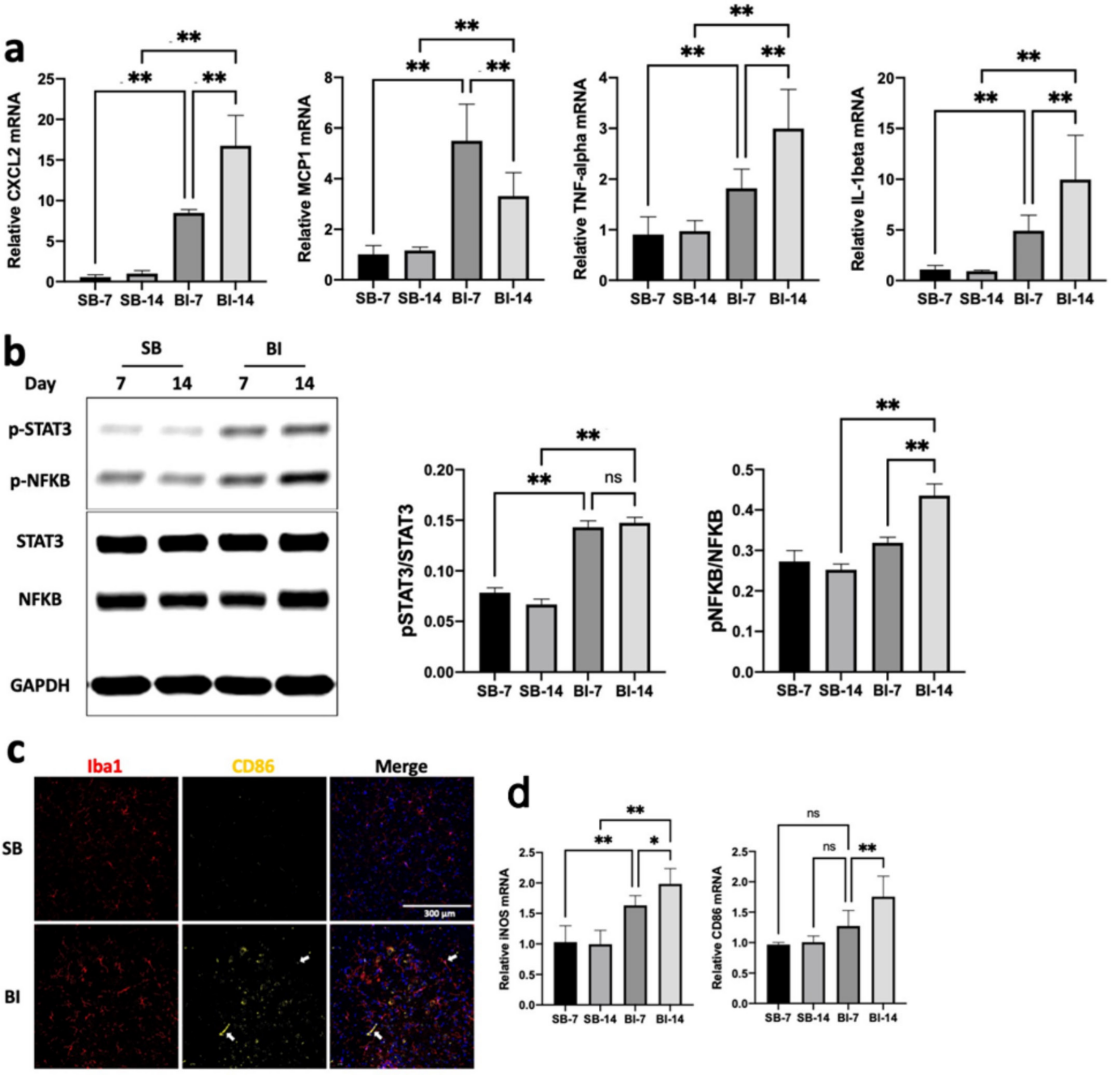
**BI leads to inflammatory chemokines cytokines, and M1 macrophage phenotype in the ventral horn of spinal cord. a:** Quantitative PCR mRNA expression of chemokines (CXCL2 and MCP1) and cytokines (IL‐1β, TNFα) were measured and normalised to GAPDH. The mRNA expression of pro‐inflammatory and chemokine molecules, IL‐1β (*p* = 0.0003, *p* < 0.0001), TNF‐α (*p* = 0.0057, *p* < 0.0001), and CXCL2 (*p* < 0.0001, *p* < 0.0001), MCP‐1 (*p* < 0.0001, *p* = 0.0023), was significantly increased at day 7 and/or 14 post‐injury in BI relative to SB (*p*‐values in the parenthesis in the order of day 7 and 14, respectively). **b**: Immunoblots of lumbar spinal cord (L3‐L4 segments) isolated from BI and SB mice at day 7 and 14 post‐injury probed for STAT3 and NFκB and normalised to GAPDH as loading control and fold change of the immunoblot was determined using Image J to measure band intensities of target protein to GAPDH (left panel). Immunoblot was quantified using Image J to measure band intensities of target proteins (phosphorylated vs. total molecule, right panels). The phosphorylated STAT3 (*p* < 0.0001, *p* < 0.0001) at day 7 and 14 and phosphorylated NFκB at day 14 (ns: *p* = , *p* < 0.0001), markers of upstream inflammatory signal transduction proteins, were significantly upregulated in BI. **c**: Lumber spinal cord samples (L3‐L4 segments) were cryo‐sectioned for the analysis of microglia phenotypes by immunofluorescent staining against the pro‐inflammatory marker, CD86 (classical M1 subtype, yellow staining in the left panel with white arrows pointing to microglia co‐stained by Iba1 and CD86). Compared to SB, BI showed increased numbers of CD86 positive cells; some of them colocalized with Iba1 positive microglia (indicated by the white arrows of merged Figure 2 c). **d**: RT‐PCR for transcripts of pro‐inflammatory microglia subtypes (iNOS and CD86 for classical M1 phenotype) were performed on the ventral horn of lumber spinal cord and shown by normalising to the internal control, GAPDH. BI increased iNOS at day 7 (*p* = 0.0003) and day 14 (*p* < 0.0001) and CD86 (*p* < 0.0001) at day 14. The transcripts increased in a time‐dependent manner at 7 towards day 14 (*p* = 0.024, 0.0012, and 0.0003 for iNOS, and CD86, respectively). Data are means ± SD; *n* = 5–8, *p < 0.05, **p < 0.01.

In the following studies, we examined if the microgliosis induced by BI leads to microglia activation evidenced as pro‐inflammatory cytokine and chemokine release; the release of inflammatory cytokines can lead to neuronal damage including motor neuron loss as shown in pathologic state of encephalitis, ALS and other motor neuron diseases [18, 19, 20, 21, 22]. The microgliosis‐mediated cytokine and chemokine release was, therefore, measured by RT‐PCR and included pro‐inflammatory IL‐1β, TNF‐α, CXCL2 and MCP‐1(a.k.a., CCR2) at day 7 and 14 after BI. All cytokines and chemokines enumerated above were significantly upregulated at day 7 and day 14 (CXCL2: *p* < 0.0001, *p* < 0.0001, MCP1: *p* < 0.0001, *p* = 0.0023, TNF‐α: *p* = 0.0057, *p* < 0.0001, IL‐1β: *p* = 0.0003, *p* < 0.0001, for day7 and day14, respectively, Figure 2a). Additionally, innate immune inflammatory‐transducer‐proteins, pSTAT3 and pNF‐κB, were significantly upregulated at both 7 and 14 days after BI (Figure 2b). Furthermore, the microglia expression of CD86, a pro‐inflammatory M1 phenotype marker (Figure 2c), was increased in the ventral horn of BI mice in a time‐dependent manner along with increased transcripts both CD86 and iNOS, both pro‐inflammatory markers of M1 phenotype (Figure 2d).

### 3.3 Burn Injury Induces Neuromuscular Junction (NMJ) or Synaptic Denervation and Muscle Mass Loss

**FIGURE 3:**
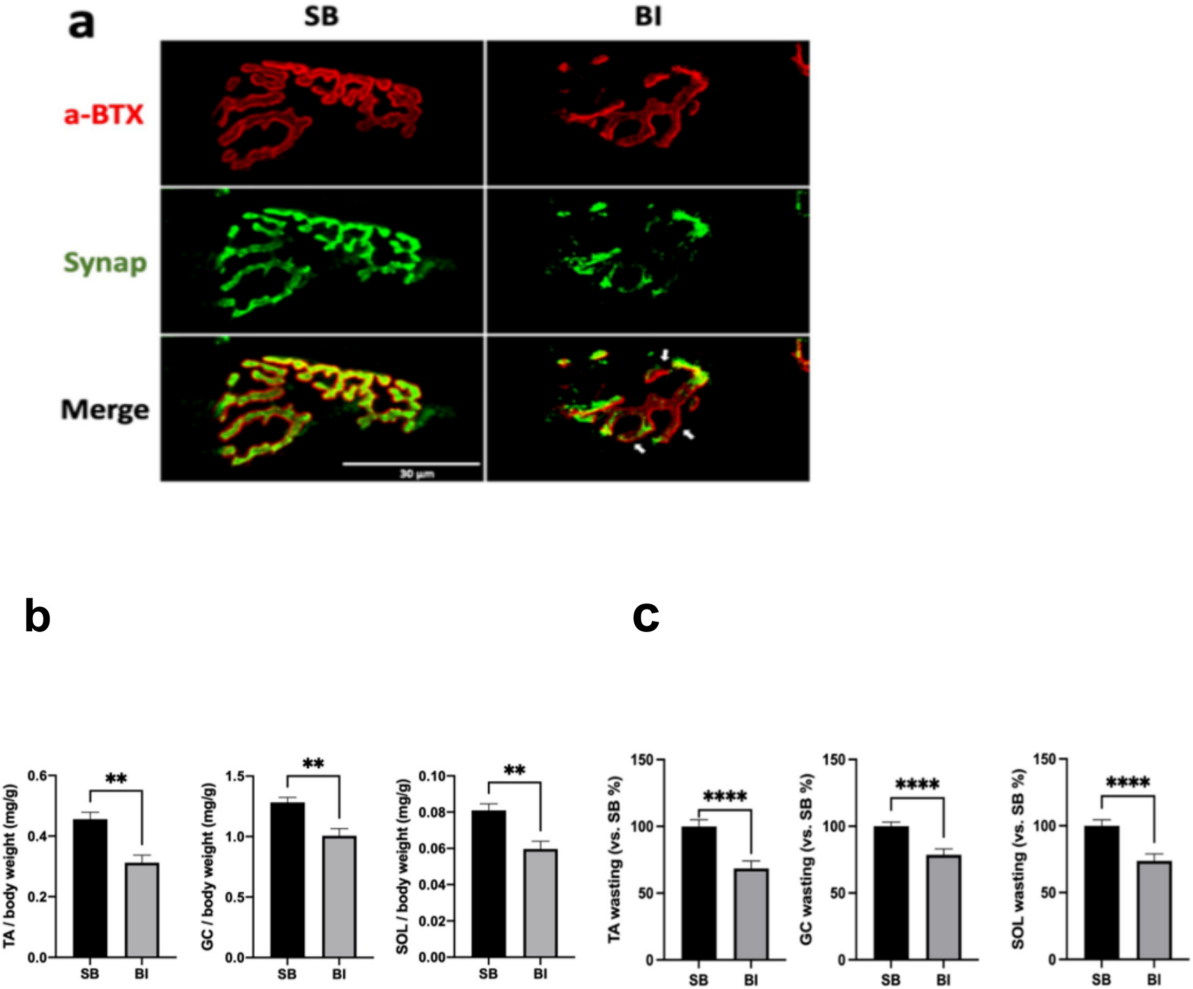
**Burn injury induced neuromuscular synapse disintegration and muscle wasting. a**: Tibialis anterior muscles were stained with α‐BTX (red) to detect acetylcholine receptors at the post‐synaptic membrane of the muscle. Presynaptic nerve terminal neurons were stained by anti‐synaptophysin antibody (green). The endplates in the SB group had a smooth and pretzel shaped appearance. The merged image shows perfect apposition of the pre‐and post‐synaptic nerve and muscle, respectively, suggesting normal undisturbed innervation of the synapse in SB. In the BI mice, the normal pretzel‐like shaped appearance was lost, and the endplate appeared fragmented with irregular edges. The presynaptic nerve component (green) was partially lost in BI, leading to an imperfect apposition to muscle (pointed by white arrows), suggesting partial denervation in BI. (Scale bar = 30 μm). **b**: The changes in muscle weight in SB, and BI group mice at day 14. Dry muscle weights of TA, SOL, and GC muscle) are expressed as a ratio to body weight pre‐BI or SB. The body weight at day 0 was used in view of body weight loss induced by BI at day 14. Those histograms show that tibialis anterior, gastrocnemius and soleus muscles masses were decreased by 31.7%, 23.4%, and 27.5%, respectively, at day 14 after BI. Data are means ± SD; *n* = 5–8 mice/group. **p < 0.01. **c**: The figure shows the absolute weights of tibialis, gastrocnemius and soleus relative to body weight. All three muscles had a significant decrease in muscle mass measured mg/g.

To explore if MNL observed in the ventral horn of BI mice has distant downstream effects, the morphology of NMJ (synapse) was examined. In SB mice, the NMJ in the tibialis anterior muscle showed the typical pretzel‐like shape with smooth boundary, suggesting the normal aggregates of acetylcholine receptors (Figure 3a, labelled by α‐bungarotoxin). The presynaptic nerve terminal stained with synaptophysin showed good apposition of nerve to muscle AChRs confirming normal innervation (merged Figure 3a bottom). After BI, however, the muscle and nerve (synapse) margins disintegrated and fragmented with irregular edges. In some parts of the NMJ, staining of presynaptic nerve terminal was absent suggesting partial denervation in BI. In the SB mice, the area of post‐synaptic AChR occupancy (stained with α‐BTX) closely matches the area of presynaptic nerve terminals (stained with synaptophysin), with an occupancy rate of 71.8%. In contrast, BI mice exhibit a significantly reduced occupancy rate of 39.6% of the nerve terminal with the AChRs. These changes in NMJ morphology indicated that BI caused disruption of the NMJ or synapse, mimicking a denervation state which could have implications for anterograde and retrograde signals to and from muscle to nerve and *vice versa*. [11, 12] Further the downstream effects of MNL and synaptic disruption on muscle were next examined by assessing muscle mass in the tibialis anterior (TA), soleus (SOL), and gastrocnemius (GC) at day 14 after BI. To exclude the effects of edema on muscle mass, the dry muscle weight was measured relative to pre‐burn body weight. Compared with SB group, the muscle mass of the TA, SOL, and GC normalised to pre‐burn body weight were significantly (Figure 3b). Conversion of the muscle masses relative to SB controls, the TA, SOL, and GC were decreased 31.7%, 23.4%, and 27.5%, respectively at day 14 after BI (Figure 3c).

### 3.4 GTS‐21 Mitigates Microgliosis and Inflammatory Phenotype in BI WT but Not in the A7KO Mice

**FIGURE 4:**
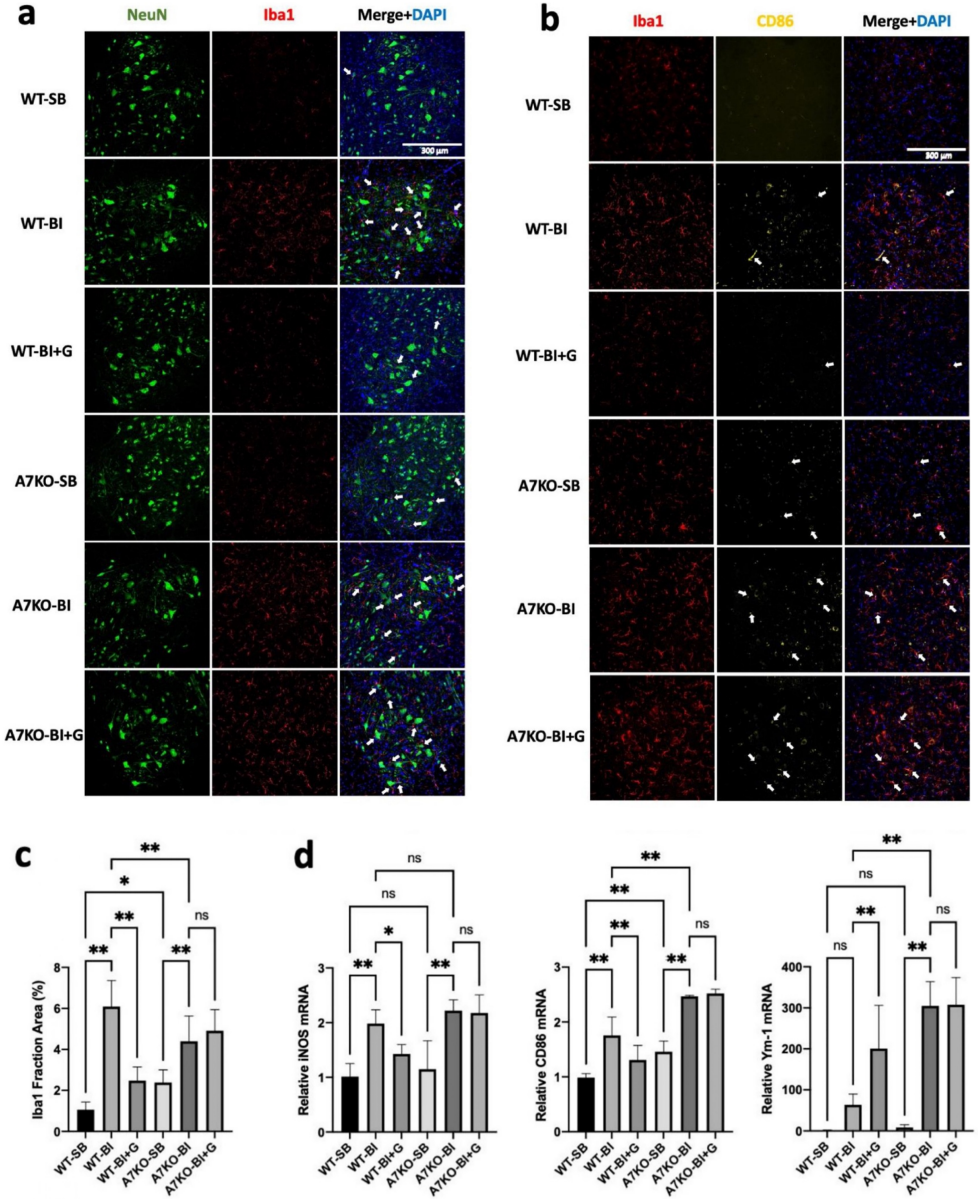
**Co‐staining of neurons, microglia and nuclei after Burn injury with or without GTS‐21 in wild type and A7KO (α7 AChR) knockout mice. a**: At day 14 after BI or sham‐burn (SB), lumbar 3–4 spinal cord ventral horn segments were cryo‐sectioned and stained for motor neurons (NeuN, green), microglia (Iba1, red), and nuclei (DAPI, blue). Ventral horn motor neurons (diameter ≥ 25 μm) were detected as green staining. Similar to observations in Figure 1, BI induced microgliosis in the ventral horn. There were increased numbers of microglia interacting with neurons in the ventral horn in BI, as compared to SB (pointed by white arrows in the merged figure for Iba1 and NeuN). Microgliosis and the increased interaction between microglia and neurons were mitigated by GTS‐21 treatment in the wild type mice (WT). Compared to wild type SB, the α7 knockout mice with SB showed a higher (84%) microgliosis. The beneficial effects of GTS‐21 against BI were not observed in the α7 knockout (A7KO) mice. This suggests that even in the basal uninjured state the α7AChRs have a role in spinal homeostasis. **b**: At day 14 after BI, the ventral horn of the lumber spinal cord was co‐stained with microglia marker, Iba1 (red), and pro‐inflammatory CD86 (yellow) expressed in microglia and monocytes/macrophages. Areas for double‐positive cells, suggestive of the pro‐inflammatory (classically M1) microglia phenotype, are pointed by white arrows. **c:** Quantification of microglia stained by Iba1 in the ventral horn, is shown as a bar graph based on image segmentation and the cell area size measurement (200X magnification) to confirm the findings in Figure 4a. BI caused as significant increase in microgliosis, which was normalised by GTS‐21 in wild type but not in the A7KO mice. **d:** Transcripts of markers of microglia pro‐inflammatory phenotype iNOS and CD86, and anti‐inflammatory, Ym‐1 were quantified in the lumbar spinal ventral horn by RT‐PCR and normalised to the internal control, GAPDH. In the WT, BI caused increase of pro‐inflammatory markers (iNOS and CD86), but not of anti‐inflammatory marker (Ym‐1). GTS‐21 treatment mitigated the increment of pro‐inflammatory microglia in the wild type mice with BI, but not in the A7KO mice. GTS‐21 treatment increased the expression of the alternatively activated Ym‐1 of wild type BI mice, reflecting M2 phenotype. Basal level of pro‐inflammatory marker (CD86) was high in the A7KO mice than WT (A7KO‐SB vs. WT‐SB), which was exacerbated by BI (A7KO‐BI vs. WT‐BI). Both pro‐ and anti‐inflammatory markers (CD86 and Ym‐1, respectively) were higher in the A7KO‐BI than in WT‐BI. The marker for anti‐inflammatory subtype of microglia, Ym‐1 was increased by GTS‐21 treatment in the WT‐BI animals, but not in A7KO mice with BI. *p < 0.05, **p < 0.01.

Previous studies have documented the expression of α7AChR on the microglia and stimulation of α7AChR leads to mitigation of inflammatory cytokine and chemokine release [13, 14, 26]. GTS‐21, a selective α7AChR agonist, was administered in vivo to exploit the anti‐inflammatory properties exhibited by α7AChRs expressed in microglia. GTS‐21 effects on microglia cytokines and chemokines, MNL and nuclear changes were examined in the ventral horn. In order to minimise rodent numbers, the effects of GTS‐21 were tested in wild type and A7KO mice with and without BI only. (The effects of GTS‐21 on WT‐SB and A7KO were not performed). As previously described in Figure 1, BI caused spinal microgliosis compared to SB; the administration of GTS‐21, however, reduced the BI‐induced microgliosis in the wild type mice (Figure 4a). The microgliosis was more significant (*p* < 0.05) in the α7KO mice even with SB (126% more than WT‐SB). BI‐associated increased protein expression of inflammatory markers and CD86 was suppressed by GTS‐21 (Figure 4b). The microgliosis with BI was further exaggerated with BI to A7KO mice (84.4% increase from A7KO‐SB) and was unchanged by GTS‐21 treatment (Figure 4c). The succeeding studies examined if the mitigation of microgliosis with GTS‐21 was associated with suppression of inflammatory phenotype. Consistently, the increased transcriptional expression of iNOS, and CD86 were significantly mitigated by GTS‐21 (Figure 4d). The marker for anti‐inflammatory subtype of microglia, Ym‐1 [27] was increased by GTS‐21 treatment in the WT‐BI animals; GTS‐21 did not change the expression of Ym‐1 in A7KO mice with BI (Figure 4d).

### 3.5 Acute Anti‐Inflammatory Effects of GTS‐21 on Spinal Inflammatory Responses

**FIGURE 5:**
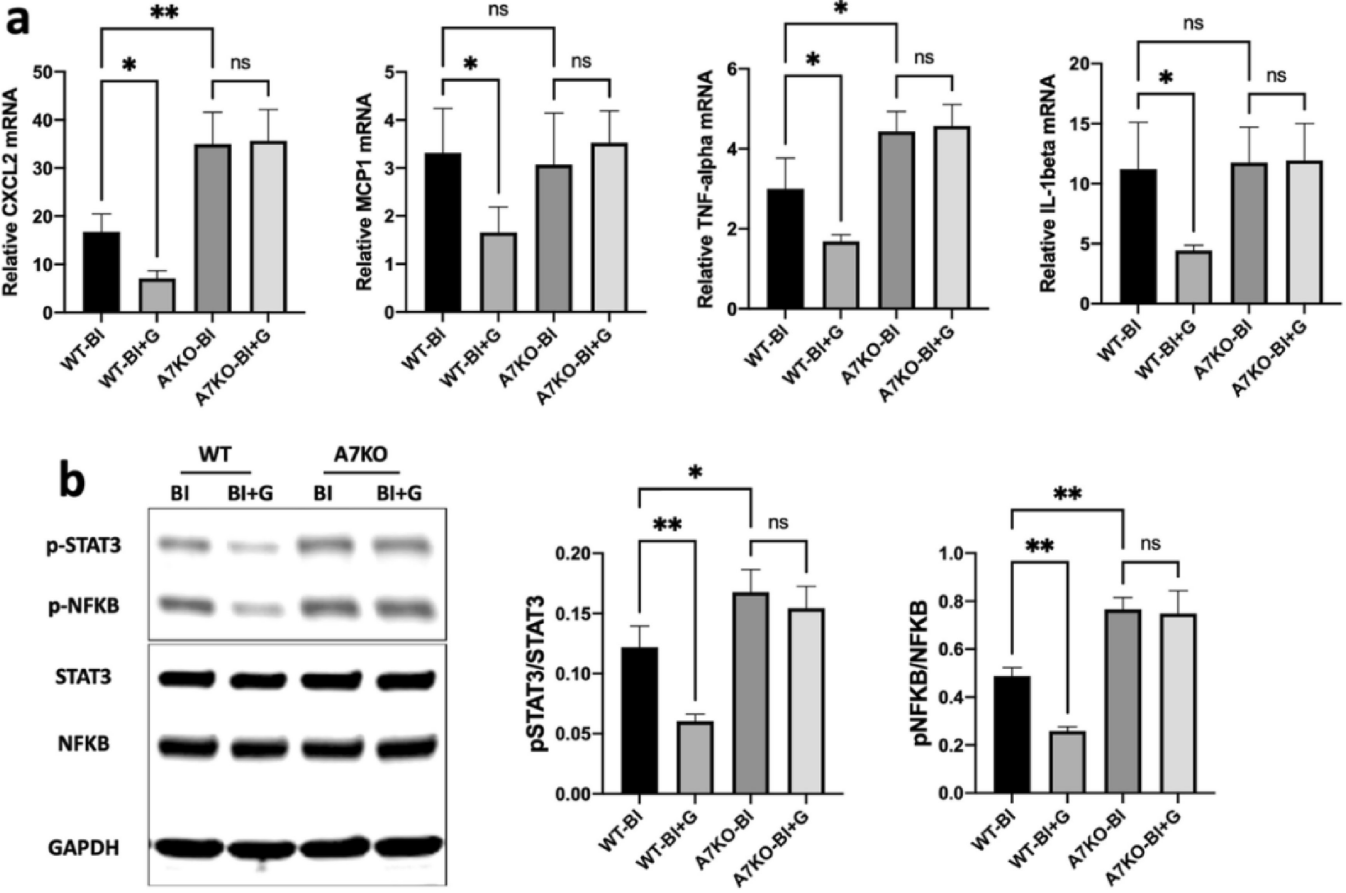
**Anti‐inflammatory effect of α7 AChR stimulation against BI induced inflammatory response in the spinal cord. a**: Quantitative PCR mRNA expression of pro‐inflammatory cytokines (IL‐1β, TNFα) and chemokines (CXCL2 and MCP1) were measured and normalised to GAPDH in the ventral horn of the spinal cord samples from BI mice with or without GTS‐21 treatment. We showed that the mRNA expression of pro‐inflammatory and chemokine molecules, IL‐1β, TNF‐α, and CXCL2, MCP‐1 were significantly (*p* < increased compared to SB at day 7 and/or 14 post‐BI). In these experiments, the effects of GTS‐21 were studied only in BI WT and A7KO mice. The administration of GTS‐21 significantly ameliorated inflammatory chemokine and cytokine expression in the wild type mice (WT). The beneficial effect of GTS‐21 was, however, nullified in the α7 AChR knockout mice (A7KO). **b**: the immunoblots of lumbar spinal cord (L3‐L4 segments) isolated from BI mice at day 7 and 14 post‐injury showed increased STAT3 and NFκB expression when normalised to GAPDH as loading control. In the following experiments, the effects of GTS‐21 inflammatory proteins were evaluated in BI WT and KO mice only were tested: The probed immunoblots were quantified to measure band intensities of target proteins (phosphorylated vs. total molecule). The increased levels of the phosphorylated STAT3 and of phosphorylated NFκB of BI were significantly reduced by GTS‐21 treatment in the wild type (‘WT’). GTS‐21 treatment of BI A7KO mice showed no mitigation of the STAT3 and NFkB phosphorylation. Data are means ± SD; *n* = 5–6, for BI and SB, * *p* < 0.05, **p < 0.01.

These experiments explored further if the beneficial effects of GTS‐21 affected the inflammatory cytokines and the other inflammatory transducer proteins. Quantitative RT‐PCRs for mRNA expression in the ventral horn were performed and normalised GAPDH in WT‐BI and A7KO with and without GTS‐21 at day 14. The administration of GTS‐21 significantly ameliorated inflammatory cytokine and chemokine expression of IL‐1β, TNF‐α, and CXCL2, MCP‐1, respectively, in the wild type mice (Figure 5a). The beneficial effect of GTS‐21 was, however, nullified in the A7KO mice. Immunoblots of lumbar spinal cord (L3‐L4 segments) isolated from BI mice were probed for STAT3 and NF‐κB and normalised to GAPDH as loading control. The probed immunoblots were quantified to measure band intensities of target proteins (phosphorylated vs. total molecule). The increased levels of the pSTAT3 and of pNF‐κB of BI were significantly reduced by GTS‐21 treatment in the wild‐type (WT) mice. GTS‐21 treatment of BI A7KO mice showed no mitigation of the STAT3 and NF‐kB phosphorylation indicating the specificity of GTS‐21 (Figure 5b,c).

### 3.6 GTS‐21 Alleviates BI‐Induced Motor Neuron Loss and Neuronal Apoptosis

**FIGURE 6:**
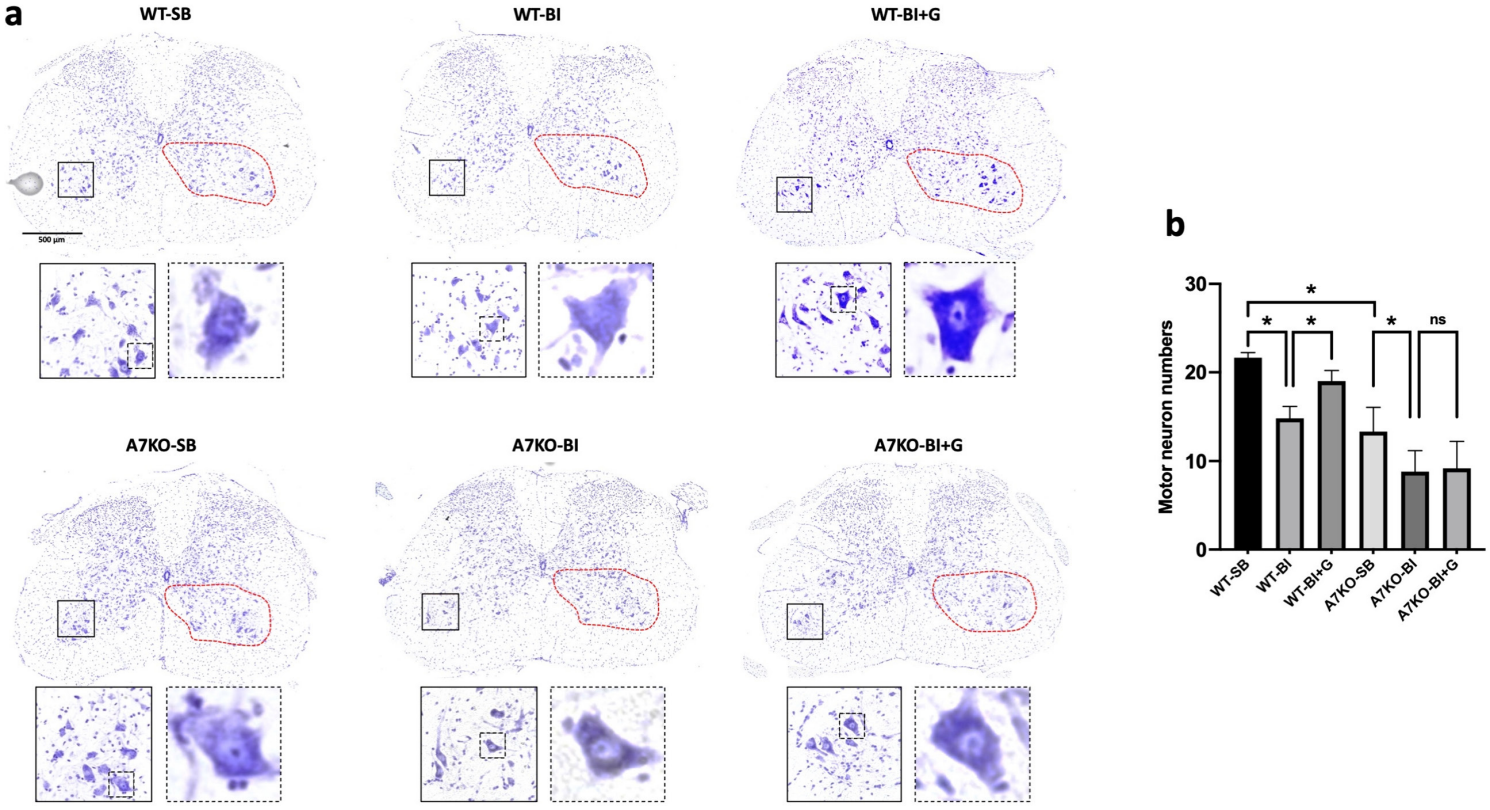
**Motor neuron numbers quantified by Nissl staining. a**: Nissl staining of spinal cord dorsal and ventral horn of the Sham Burn (SB) and burn injured (BI) mice with or without GTS‐21 treatment were examined in wild type (WT) or α7 AChR knockout (A7KO) mice. In the WT mice with SB (WT‐SB), the typical dark blue staining of the cytoplasmic portion due to Nissl bodies was observed (see magnifications of the ventral horn area from the insets enclosed by solid black line, and for the representative neurons from the insets enclosed by dotted line). In the WT‐BI mice, Nissl body was less obvious with the lighter staining of the cytoplasm and the overall cell shape swollen in BI with no GTS‐21. BI decreased the motor neuron numbers compared to WT SB (31.6% reduction). GTS‐21 ameliorated the motor neuron loss of in the ventral horn of the WT mice (improved to 12.3% reduction from SB), but not in the A7KO mice. Note that in the SB A7KO mice, there was decreased neuron numbers compared to SB WT (61.5% of WT‐SB) and the BI exacerbated the neuronal loss in the A7KO mice. **b**: The motor neuron numbers (diameter ≥ 25 μm, 200X magnification) in the ventral horn of each group were quantified. BI caused a significant decrease in neuron numbers, which was significantly mitigated by GTS‐21. There was significant neuron loss in the SB A7KO compared SB WT; the presence of BI aggravated the neuron loss in the A7KO. Data are means ± SD; *n* = 6. *p < 0.05.

Microglia activation with its concomitant cytokine release results in deleterious effects on the motor neuron in many neurological diseases including amyotrophic lateral sclerosis and Parkinson's disease [17, 18]. In the following studies, the anti‐inflammatory properties exhibited by α7AChRs in microglia were capitalised using GTS‐21 as agonist to mitigate microglia and neuronal changes. Nissl‐stained spinal cord dorsal and ventral horn of the SB and BI mice with or without GTS‐21 were examined in wild type or A7KO mice. In the ventral horn of the wild‐type SB mice, the typical dark blue staining of the peripheral cytoplasmic portion due to the presence of Nissl bodies was observed (Figure 6a, inset below‐ 400X magnification**)**; in the wild type with BI without GTS‐21 treatment, the spinal cord central motor neuron nucleus was lightly stained and less obvious, the overall cell shape was swollen and the clear cytoplasm around the nucleus was absent (Figure 6b‐ boxed inset 400X magnification). The numbers of morphologically normal motor neurons were fewer (31.6% reduction compared to SB, satisfying threshold criteria of 25 μm or higher). GTS‐21 treatment partially reverted these pathologic changes (improved to 12.3% reduction from SB, central nucleus was more visible together with the central cytoplasm Figure 6b). Of note, the Nissl‐stained spinal cord of A7KO mice with SB showed reduced motor neuron numbers (61.5% of WT even with SB). BI exacerbated the pathologic morphology in the A7KO mice (33.8% reduction from A7KO‐SB, Figure 6a). The favourable effects of GTS‐21 were not observed in the A7KO mice suggesting an important role of α7AChRs in the preservation and/or reversal of the damaging effects of microglia on neurotransmission from motor neuron to muscle.

**FIGURE 7:**
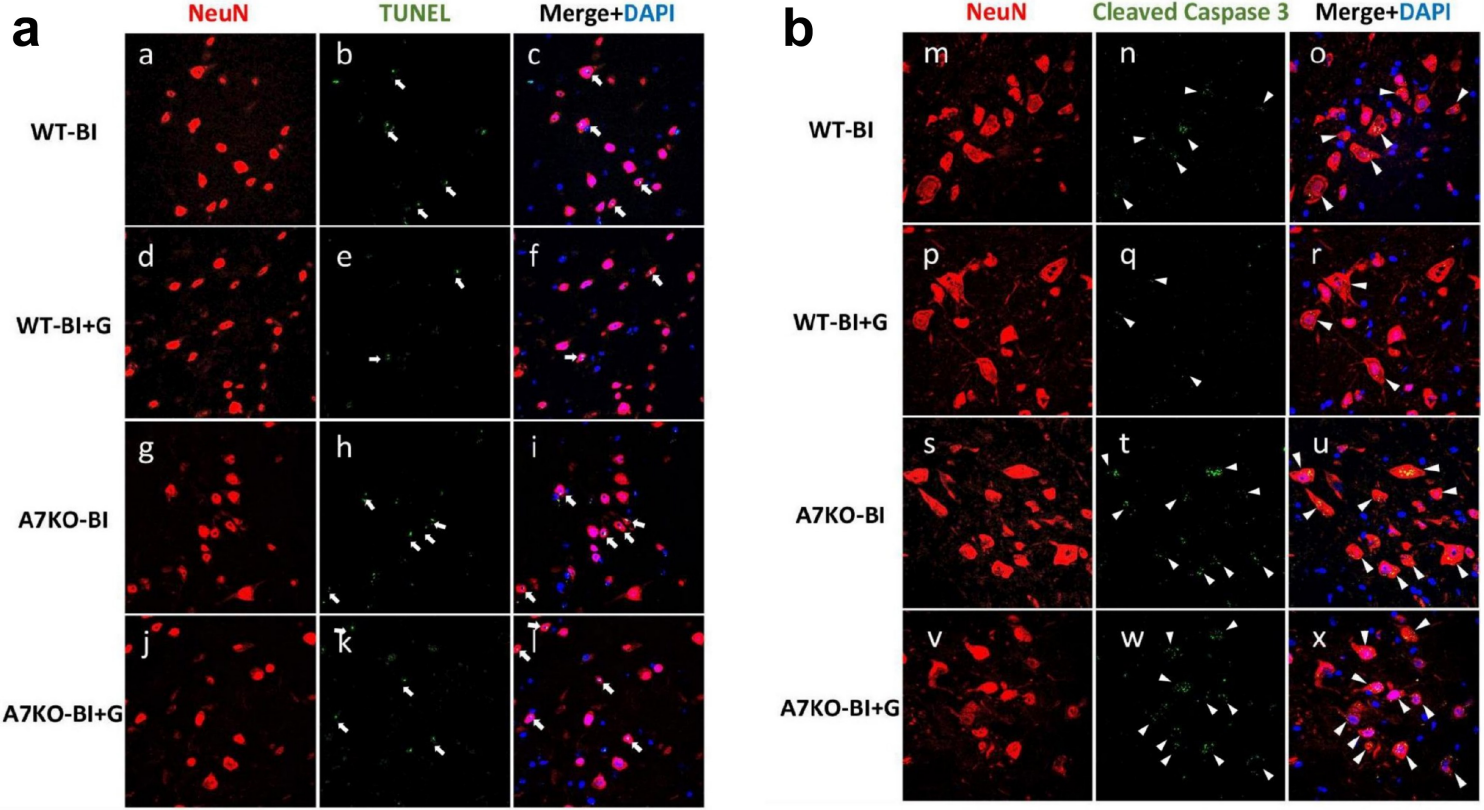
**GTS‐21 prevents motor neuron loss by blocking neuronal apoptosis**. **a**: In situ TUNEL assay for apoptosis (green) in the ventral horn area of the spinal cord with counter‐staining for neurons using NeuN (NeuN, red) and nuclei (DAPI, blue) in the burned injured (BI) and sham‐burn (SB) mice with or without α7AChR agonist, GTS‐21. As compared to BI without treatment, GTS‐21 treatment reduced the TUNEL positive nuclei in the neurons in the wild type mice. In the α7AChR knockout mice, however, the therapeutic effect by GTS‐21 was not observed. **b**: Immunostaining against activated caspase‐3 (green) of the ventral horn area is shown with counterstaining against neurons (NeuN, red) and nuclei (DAPI, blue). Compared to BI group without treatment, GTS‐21 administration lowered the expression of caspase‐3. The therapeutic effect by GTS‐21 was nullified in α7AChR knockout mice.

Next, to understand the mechanisms of MNL and its reversal by α7AChR stimulation by GTS‐21, the extent of neuronal apoptosis in the ventral horn was analysed using in situ TUNEL assay. GTS‐21 treatment reduced the extent of neuronal cell death in WT mice, but not in A7KO mice (Figure 7a). Similarly, cleaved caspase‐3, as shown by the immunofluorescent staining showed suppression of caspase‐3 activation by GTS‐21 treatment in the WT, but not in A7KO mice (Figure 7b).

### 3.7 Prevention of Motor Neuron Loss by GTS‐21 Mitigates NMJ Disintegration and Muscle Wasting

**FIGURE 8:**
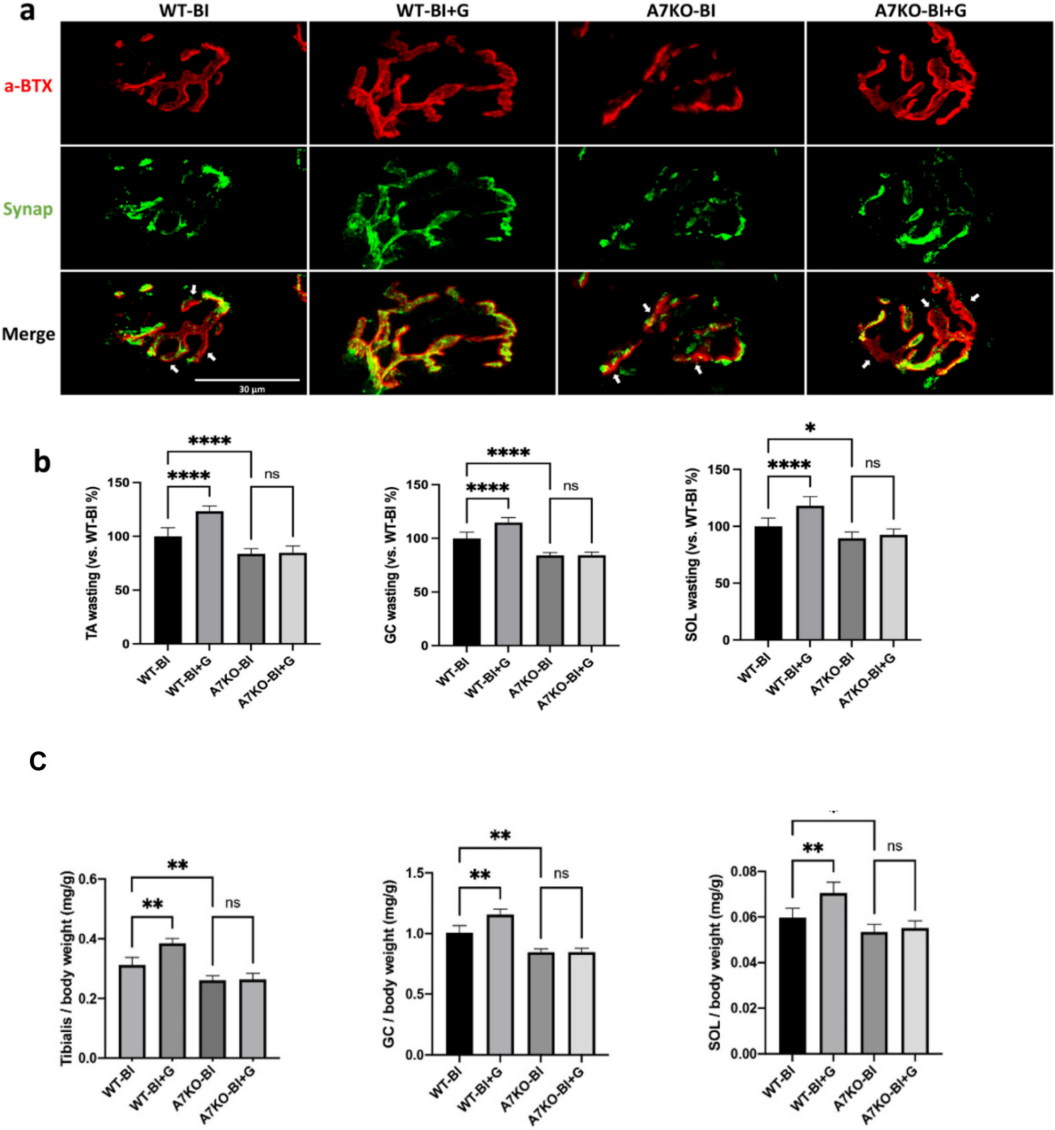
**GTS‐21 stimulation of** α**7 AChR maintains synaptic integrity and mitigates muscle wasting. a**: The morphology of synaptic membrane and the apposition of pre‐ and post‐synaptic apparatus was compared in BI mice with or without α7AChR agonist, GTS‐21 (‘+G’) in WT and α7AChR knockout (‘A7KO’) mice. Tibialis anterior muscles of BI mice were stained with α‐BTX (red) for post‐synaptic muscle AChRs on muscle membrane and anti‐synaptophysin antibody for presynaptic neuron to monitor the disintegration of the synapse denervation status, with or without GTS treatment. The disintegrated AChR morphology (red) and the partial denervation phenotype (poor apposition of a‐BTX and synapse, pointed by white arrows) in the BI group was improved by GTS‐21 treatment in the wild type mice (‘WT‐BI+G’). The therapeutic efficacy of GTS‐21 was lacking in α7AChR KO mice. (Scale bar = 30 μm). **b**: The changes in muscle weight of SB, and BI group mice at day 14 in the WT or α7AChR KO mice. Dry muscle weights TA, SOL, and GC muscle] are expressed as a ratio to body weight pre‐burn. The body weight at day 0 was used in view of body weight loss induced by BI at day 14. Muscle wasting by BI was significantly improved by GTS‐21 treatment in the WT, but not in α7AChR KO mice. Data are means ± SD; *n* = 5–8 mice/group. *p < 0.05, **p < 0.01. **c.** The figure shows the absolute weights of tibialis, gastrocnemius and soleus relative to body weight. All three muscles had a significant decrease in muscle mass measured mg/g.

These experiments explored the downstream effects of prevention of MNL by GTS‐21 treatment on both the synaptic denervation and MW induced by BI. As compared to BI mice without treatment, GTS‐21 rescued the disintegration of the endplate, and mitigated the partial denervation in the WT mice, but not in the A7KO mice. In wild‐type BI mice not treated with GTS‐21, the area of presynaptic nerve terminals (stained with synaptophysin) was reduced relative to the area of postsynaptic AChR occupancy (stained with α‐BTX), indicating partial denervation (occupancy rate: 27.4%). Treatment with GTS‐21 significantly ameliorated this denervation, restoring occupancy to 71.3%. However, in A7KO knockout mice, the reduced occupancy observed after burn injury (31.4%) showed no significant improvement following GTS‐21 treatment (35.8%) (Figure 8a). It should be noted that a 70% occupancy in terms of area size comparison corresponds to near‐perfect apposition of the pre‐ and postsynaptic apparatus, as postsynaptic receptors typically occupy a larger anatomical area because AChRs extend to the primary and secondary clefts on the muscle.

Furthermore, muscle weights of tibialis, soleus and gastrocnemius were significantly increased with GTS‐21 treatment of the wild‐type BI mice, but not in A7KO mice (Figure 8b). In other words, the treatment of BI wild type mice with GTS‐21 significantly increased the muscle of tibialis, gastrocnemius and soleus muscle weights by 25%, 15%, and 20%, respectively (Figure 8c). These results indicate that GTS‐21 mitigates microglia‐mediated neuroinflammation and reduced MNL by apoptosis, which in turn had beneficial effects on the synaptic denervation and MW changes of BI (Figure 8a‐c).

## 4 Discussion

The salient findings of this study are that: 1) BI leads to microgliosis together with ventral horn MNL due to apoptosis mediated at least in part by caspase‐3 activation (Figure 1); 2) The microgliosis and microglia activation of BI increased the expression of transcripts of pro‐inflammatory markers IL‐1β, TNF‐α, CXCL2 and MCP‐1 (a.k.a., CCR2), CD86, and iNOS and protein expression of pSTAT3 and pNF‐κB, reflecting the inflammatory status of the spinal microglia (Figure 2); 3) synaptic disintegration mimicking denervation and muscle protein loss occurred in hind limb muscles distant from body burn (Figure 3); 4) GTS‐21 decreased microgliosis and microglia activation exemplified by the decreased expression of inflammatory markers enumerated above; 5) GTS‐21 decreased pro‐inflammatory responses and increased the anti‐inflammatory marker Ym‐1 (Figure 4); 6) Neuronal apoptosis was also mitigated by GTS‐21 (Figure 7); 7) The typical dark blue staining of the cytoplasm due to Nissl bodies in motor neurons was less obvious due to lighter staining and cell was swollen in WT‐BI mice (Figure 6). Neuron numbers were also decreased (31.6% reduction compared WT‐SB mice, Figure 6). GTS‐21 ameliorated the ventral horn neuron loss in the WT mice (improved to 12.3% reduction from untreated WT‐BI); 8) GTS‐21 ameliorated synaptic disintegration and muscle mass loss (Figure 8). The beneficial anti‐inflammatory effects of GTS‐21 were absent in the A7KO mice.

Most previous studies on MW of BI, focusing on reversal of the muscle hyper‐catabolic and hypo‐anabolic state and/or providing enhanced nutritional/anabolic supplements, have not rectified the MW of BI [5, 6, 7, 8]. Ma et al., previously provided evidence that microglia activation and MNL but their relationship to synaptic disintegration and MW was not provided [15]. The current studies using GTS‐21 to take advantage of the anti‐inflammatory properties exhibited by α7AChRs expressed by immune microglia cells and demonstrated that GTS‐21 decreased microglia activation led to mitigated MNL, which in turn alleviated the synaptic disintegration and muscle mass changes. Since α7AChRs are expressed in macrophages too, it is possible that some of the improvements in muscle mass could be attributed to anti‐inflammatory properties exhibited by macrophages directing on muscle. However, the decreased MNL with GTS‐21 cannot be attributed to decreased macrophage inflammation but attributable to the decreased microglia activation by GTS‐21. This reveals a cause‐and‐effect relationship between decreased the spinal inflammatory proteins and improved motor neuron changes. The fact that even in the non‐injured (SB) state, the MNL and muscle mass was lower in the A7KO mice relative to age‐matched non‐BI wild type mice suggest that even in the basal non‐injured (burned) state, the α7AChRs have a protective role in the maintenance of spinal homeostasis. One may pose the question as to why there was no overt evidence of muscle weakness in A7KO mice in the basal or BI injured despite the MNL and muscle wasting. This is most likely related tremendous margin of safety of neuromuscular transmission. This scenario is akin to that seen early in the aged where motor neuron changes and denervation occurs but muscle wasting is not evident till late in the disease [28].

A fraction of CD86 positive cells in the spinal cord were Iba1‐negative (Figure 2c); infiltrating macrophages/monocytes could account for this and contribute augmented spinal neuroinflammation of BI. Furthermore, the circulating innate immune macrophages also constitutively express α7AChRs. Our studies presented here do not distinguish GTS‐21 effects on the circulating inflammatory macrophages. The cross talk between systemic macrophages and muscle has also received minimal attention both in BI and other MW conditions and needs exploration. Future studies [29, 30] could verify the pivotal roles of macrophages vs. microglia in the spinal cytokine, neuronal and muscle changes.

The altered inflammatory phenotype of microglia was manifested by the significantly upregulated transcript‐markers of inflammation (Figure 2). This inflammatory soup probably affects neuronal homeostasis and ultimately even survival as demonstrated in many neuropathologic states such as ALS, encephalitis, and Alzheimer's disease where neuro‐inflammation also leads to muscle changes [31, 32, 33]. The microglia‐mediated MNL after BI may explain the well documented phenomenon that even 5 years after BI, despite continued anabolic therapy and exercise, the MW of BI has not been reversed to normal [2, 3, 4, 5, 6, 7, 8].

In the normal mature muscle, in addition to anterograde signals from motor neuron to muscle, muscle factors acting retrograde induce differentiation signals to the presynaptic motor axon to maintain not only synaptic and nerve integrity but also motor neuron stability [34, 35, 36]. In some disease states (e.g., ALS) a significant increase in the expression of inflammatory markers (CD11b and CD68) and inflammatory cytokines (IL‐1β and TNF‐α) was observed in muscle together with degenerative processes in the neuromuscular junctions very early in disease development [37]. Concordantly, interleukin‐6 (IL‐6) release effecting bone morphogenic protein (BMP) function leads to loss of protective muscle to nerve signals with denervation and MW in cancer [12, 38]. These lines of evidence suggest that BI, cancer and neurodegenerative diseases share some common traits regarding synaptic denervation and MW and support the mutual dependency of nerve terminal and skeletal muscles for maintenance of their integrity involving anterograde and retrograde trophic signalling. Thus, it is possible that some of the synaptic changes are related to direct effects of systemic inflammation on muscle; a contributory direct effect of systemic inflammation on muscle to cause the synaptic disintegration cannot, therefore, be discounted.

Our current studies demonstrate that BI‐induced systemic inflammation can lead to neuro‐inflammation and caspase‐3 mediated apoptotic MNL, which affects distant muscles. The stimulation of α7AChRs by selective agonist, GTS‐21, ameliorates not only the spinal inflammatory changes and MNL by decreasing cleaved caspase‐mediated apoptosis, but also improves synaptic denervation and MW. These beneficial effects by GTS‐21 were completely nullified in α7 AChR knockout mice, confirming the specific protective role of α7 AChR stimulation and the anti‐inflammatory therapeutic effects of GTS‐21. The α7AChR has modulatory effects even in uninjured state as demonstrated by greater spinal and muscle changes in the uninjured A7KO mice. GTS‐21 administration in BI patients may prove useful for mitigation of MW.

## Author Contributions

JY. C contributed to the design and conduction of this study, data analysis, and writing the final manuscript. Y. K. and F. X. participated in establishing the animal model and recording results. WRK provided the GTS‐21 and advised on GTS‐21 therapeutics. ZY and HL helped with spinal cord biochemical measurements and analysed the data. S. Y was involved with morphology experiments and JAJM initiated the project and continued give suggestions on next experiments. SY and JAJM read the final version of the manuscript. All authors read and approved the final manuscript.

## Conflicts of Interest

The authors declare that they have no known competing financial interests or personal relationships that could have appeared to influence the work reported in this paper.

## References

[1] F. Ravat , M. Fontaine , J. Latarjet , and D. Voulliaume , “Burn: Epidemiology, Evaluation, Organisation of Care,” La Revue du Praticien 68, no. 10 (2018): 1078–1082. 30869211

[2] E. Polychronopoulou , D. N. Herndon , and C. Porter , “The Long‐Term Impact of Severe Burn Trauma on Musculoskeletal Health,” Journal of Burn Care & Research 39, no. 6 (2018): 869–880. 30010999 10.1093/jbcr/iry035 PMC6198740

[3] J. Rinkinen , C. D. Hwang , S. Agarwal , et al., “The Systemic Effect of Burn Injury and Trauma on Muscle and Bone Mass and Composition,” Plastic and Reconstructive Surgery 136, no. 5 (2015): 612e–623e. 10.1097/PRS.0000000000001723 PMC4876821 26505718

[4] L. C. Simko , L. F. Espinoza , K. McMullen , et al., “Fatigue Following Burn Injury: A Burn Model System National Database Study,” Journal of Burn Care & Research 39, no. 3 (2018): 450–456. 28877130 10.1097/BCR.0000000000000625 PMC9218839

[5] C. Porter , D. N. Herndon , E. Borsheim , et al., “Long‐Term Skeletal Muscle Mitochondrial Dysfunction Is Associated With Hypermetabolism in Severely Burned Children,” Journal of Burn Care & Research 37, no. 1 (2016): 53–63. 26361327 10.1097/BCR.0000000000000308 PMC4691377

[6] P. T. Reeves , D. N. Herndon , J. D. Tanksley , et al., “Five‐Year Outcomes After Long‐Term Oxandrolone Administration in Severely Burned Children: A Randomized Clinical Trial,” Shock 45, no. 4 (2016): 367–374. 26506070 10.1097/SHK.0000000000000517 PMC4792676

[7] A. Abdullahi and M. G. Jeschke , “Nutrition and Anabolic Pharmacotherapies in the Care of Burn Patients,” Nutrition in Clinical Practice 29, no. 5 (2014): 621–630. 25606644 10.1177/0884533614533129

[8] M. G. Jeschke , M. E. van Baar , M. A. Choudhry , K. K. Chung , N. S. Gibran , and S. Logsetty , “Burn Injury,” Nature Reviews. Disease Primers 6, no. 1 (2020): 11. 10.1038/s41572-020-0145-5 PMC7224101 32054846

[9] C. Kim , J. Martyn , and N. Fuke , “Burn Injury to Trunk of rat Causes Denervation‐Like Responses in the Gastrocnemius Muscle,” Journal of Applied Physiology 65, no. 4 (1985): 1745–1751. 10.1152/jappl.1988.65.4.1745 3182535

[10] J. M. Ward and J. A. Martyn , “Burn Injury‐Induced Nicotinic Acetylcholine Receptor Changes on Muscle Membrane,” Muscle & Nerve 16, no. 4 (1993): 348–354. 8455647 10.1002/mus.880160403

[11] T. Kim and I. Y. Cheong , “Changes in Function and Muscle Strength of Encephalitis Survivors After Inpatient Rehabilitation,” Annals of Rehabilitation Medicine 45, no. 6 (2021): 422–430. 35000367 10.5535/arm.21133 PMC8743845

[12] R. Sartori , A. Hagg , S. Zampieri , et al., “Perturbed BMP Signaling and Denervation Promote Muscle Wasting in Cancer Cachexia,” Science Translational Medicine 13, no. 605 (2021): eaay9592. 34349036 10.1126/scitranslmed.aay9592

[13] Y. Ren , Y. Zhou , Z. You , et al., “The Nonopioid Cholinergic Agonist GTS‐21 Mitigates Morphine‐Induced Aggravation of Burn Injury Pain Together With Inhibition of Spinal Microglia Activation in Young Rats,” British Journal of Anaesthesia 129 (2022): 959–969.36243579 10.1016/j.bja.2022.07.055

[14] Y. Zhou , Y. Leung‐Pitt , H. Deng , et al., “Nonopioid GTS‐21 Mitigates Burn Injury Pain in Rats by Decreasing Spinal Cord Inflammatory Responses,” Anesthesia and Analgesia 132, no. 1 (2021): 240–252. 33264122 10.1213/ANE.0000000000005274 PMC7736563

[15] L. Ma , Y. Zhou , M. A. S. Khan , S. Yasuhara , and J. A. J. Martyn , “Burn‐Induced Microglia Activation Is Associated With Motor Neuron Degeneration and Muscle Wasting in Mice,” Shock 51, no. 5 (2019): 569–579. 30702509 10.1097/SHK.0000000000001300 PMC6537589

[16] J. L. Frost and D. P. Schafer , “Microglia: Architects of the Developing Nervous System,” Trends in Cell Biology 26, no. 8 (2016): 587–597. 27004698 10.1016/j.tcb.2016.02.006 PMC4961529

[17] L. Muzio , A. Viotti , and G. Martino , “Microglia in Neuroinflammation and Neurodegeneration: From Understanding to Therapy,” Frontiers in Neuroscience 15 (2021): 742065. 34630027 10.3389/fnins.2021.742065 PMC8497816

[18] M. Prinz , T. Masuda , M. A. Wheeler , and F. J. Quintana , “Microglia and Central Nervous System‐Associated Macrophages‐From Origin to Disease Modulation,” Annual Review of Immunology 39 (2021): 251–277. 10.1146/annurev-immunol-093019-110159 PMC8085109 33556248

[19] S. Bachiller , I. Jimenez‐Ferrer , A. Paulus , et al., “Microglia in Neurological Diseases: A Road Map to Brain‐Disease Dependent‐Inflammatory Response,” Frontiers in Cellular Neuroscience 12 (2018): 488. 30618635 10.3389/fncel.2018.00488 PMC6305407

[20] S. Hickman , S. Izzy , P. Sen , L. Morsett , and J. El Khoury , “Microglia in Neurodegeneration,” Nature Neuroscience 21, no. 10 (2018): 1359–1369. 30258234 10.1038/s41593-018-0242-x PMC6817969

[21] A. C. Wendeln , K. Degenhardt , L. Kaurani , et al., “Innate Immune Memory in the Brain Shapes Neurological Disease Hallmarks,” Nature 556, no. 7701 (2018): 332–338. 29643512 10.1038/s41586-018-0023-4 PMC6038912

[22] S. A. Wolf , H. W. Boddeke , and H. Kettenmann , “Microglia in Physiology and Disease,” Annual Review of Physiology 79 (2017): 619–643. 10.1146/annurev-physiol-022516-034406 27959620

[23] E. X. Albuquerque , E. F. Pereira , M. Alkondon , and S. W. Rogers , “Mammalian Nicotinic Acetylcholine Receptors: From Structure to Function,” Physiological Reviews 89, no. 1 (2009): 73–120. 19126755 10.1152/physrev.00015.2008 PMC2713585

[24] W. R. Kem , “The Brain alpha7 Nicotinic Receptor May Be an Important Therapeutic Target for the Treatment of Alzheimer's Disease: Studies With DMXBA (GTS‐21),” Behavioural Brain Research 113, no. 1–2 (2000): 169–181. 10942043 10.1016/s0166-4328(00)00211-4

[25] K. Takata , T. Amamiya , H. Mizoguchi , et al., “Alpha7 Nicotinic Acetylcholine Receptor‐Specific Agonist DMXBA (GTS‐21) Attenuates Abeta Accumulation Through Suppression of Neuronal Gamma‐Secretase Activity and Promotion of Microglial Amyloid‐beta Phagocytosis and Ameliorates Cognitive Impairment in a Mouse Model of Alzheimer's Disease,” Neurobiology of Aging 62 (2018): 197–209. 29175709 10.1016/j.neurobiolaging.2017.10.021

[26] M. A. Khan , M. Farkhondeh , J. Crombie , L. Jacobson , M. Kaneki , and J. A. Martyn , “Lipopolysaccharide Upregulates Alpha7 Acetylcholine Receptors: Stimulation With GTS‐21 Mitigates Growth Arrest of Macrophages and Improves Survival in Burned Mice,” Shock 38, no. 2 (2012): 213–219. 22683726 10.1097/SHK.0b013e31825d628c PMC3399057

[27] Q. Kang , L. Li , Y. Pang , W. Zhu , and L. Meng , “An Update on Ym1 and Its Immunoregulatory Role in Diseases,” Frontiers in Immunology 13 (2022): 891220. 35967383 10.3389/fimmu.2022.891220 PMC9366555

[28] S. R. Iyer , S. B. Shah , and R. M. Lovering , “The Neuromuscular Junction: Roles in Aging and Neuromuscular Disease,” International Journal of Molecular Sciences 22, no. 15 (2021): 8058. 34360831 10.3390/ijms22158058 PMC8347593

[29] A. J. Iqbal , E. McNeill , T. S. Kapellos , et al., “Human CD68 Promoter GFP Transgenic Mice Allow Analysis of Monocyte to Macrophage Differentiation in Vivo,” Blood 124, no. 15 (2014): e33–e44. 25030063 10.1182/blood-2014-04-568691 PMC4192756

[30] S. Jung , J. Aliberti , P. Graemmel , et al., “Analysis of Fractalkine Receptor CX(3)CR1 Function by Targeted Deletion and Green Fluorescent Protein Reporter Gene Insertion,” Molecular and Cellular Biology 20, no. 11 (2000): 4106–4114. 10805752 10.1128/mcb.20.11.4106-4114.2000 PMC85780

[31] S. A. Sargsyan , D. J. Blackburn , S. C. Barber , P. N. Monk , and P. J. Shaw , “Mutant SOD1 G93A Microglia Have an Inflammatory Phenotype and Elevated Production of MCP‐1,” Neuroreport 20, no. 16 (2009): 1450–1455. 19752764 10.1097/WNR.0b013e328331e8fa PMC2889291

[32] C. Parisi , G. Napoli , S. Amadio , et al., “MicroRNA‐125b Regulates Microglia Activation and Motor Neuron Death in ALS,” Cell Death and Differentiation 23, no. 3 (2016): 531–541. 26794445 10.1038/cdd.2015.153 PMC5072447

[33] A. E. Frakes , L. Ferraiuolo , A. M. Haidet‐Phillips , et al., “Microglia Induce Motor Neuron Death Via the Classical NF‐kappaB Pathway in Amyotrophic Lateral Sclerosis,” Neuron 81, no. 5 (2014): 1009–1023. 24607225 10.1016/j.neuron.2014.01.013 PMC3978641

[34] B. Berke , J. Wittnam , E. McNeill , D. L. Van Vactor , and H. Keshishian , “Retrograde BMP Signaling at the Synapse: A Permissive Signal for Synapse Maturation and Activity‐Dependent Plasticity,” Journal of Neuroscience 33, no. 45 (2013): 17937–17950. 24198381 10.1523/JNEUROSCI.6075-11.2013 PMC3818560

[35] A. P. Haghighi , B. D. McCabe , R. D. Fetter , J. E. Palmer , S. Hom , and C. S. Goodman , “Retrograde Control of Synaptic Transmission by Postsynaptic CaMKII at the Drosophila Neuromuscular Junction,” Neuron 39, no. 2 (2003): 255–267. 12873383 10.1016/s0896-6273(03)00427-6

[36] A. W. Harrington and D. D. Ginty , “Long‐Distance Retrograde Neurotrophic Factor Signalling in Neurons,” Nature Reviews. Neuroscience 14, no. 3 (2013): 177–187 . 23422909 10.1038/nrn3253

[37] J. M. Van Dyke , I. M. Smit‐Oistad , C. Macrander , D. Krakora , M. G. Meyer , and M. Suzuki , “Macrophage‐Mediated Inflammation and Glial Response in the Skeletal Muscle of a rat Model of Familial Amyotrophic Lateral Sclerosis (ALS),” Experimental Neurology 277 (2016): 275–282. 26775178 10.1016/j.expneurol.2016.01.008 PMC4762214

[38] T. A. Zimmers , “Tumours Block Protective Muscle and Nerve Signals to Cause cachexia,” Nature 598, no. 7879 (2021): 37–38. 34548663 10.1038/d41586-021-02492-9

